# SensorW: Open-Source Wear Operating System Platform for Continuous Global Positioning System and Multimodal Sensor Data Collection in Real-World Settings

**DOI:** 10.3390/s26196093

**Published:** 2026-09-25

**Authors:** Yichi Yang, Kazushige Ihara, Felipe Alfonso Sandoval Garrido, Koki Hirata, Tao Jiang, Hua Lan, Han Wu, Hongyue Yu, Yoshinori Tamada, Koichi Murashita, Ken Itoh, Kazutaka Yoshida

**Affiliations:** 1 Department of Social Medicine, Graduate School of Medicine, Hirosaki University, Hirosaki 036-8562, Japan; ihara@hirosaki-u.ac.jp (K.I.); dr_par@hirosaki-u.ac.jp (K.H.); h23gm129@hirosaki-u.ac.jp (T.J.); h23gm205@hirosaki-u.ac.jp (H.L.); h25gm122@hirosaki-u.ac.jp (H.W.); h23gm204@hirosaki-u.ac.jp (H.Y.); 2 Section of Global Health and Preventive Medicine, Global Well-being Research Institute, Hirosaki University, Hirosaki 036-8562, Japan; sandoval.felipe@hirosaki-u.ac.jp; 3 Research Institute of Health Innovation, Hirosaki University, Hirosaki 036-8562, Japan; y.tamada@hirosaki-u.ac.jp (Y.T.); murasita@hirosaki-u.ac.jp (K.M.); 4 Department of Stress Response Science, Biomedical Research Center, Graduate School of Medicine, Hirosaki University, Hirosaki 036-8562, Japan; itohk@hirosaki-u.ac.jp; 5 Institute of Global Well-being Science, Hirosaki University, Hirosaki 036-8562, Japan; ykazu123@hirosaki-u.ac.jp

**Keywords:** wearable sensing, lifelog data, mobile health

## Abstract

Digital phenotyping requires continuous, real-world collection of multimodal behavioral and physiological data. Although several smartphone-based sensing frameworks have been developed and used, open-source platforms for long-term smartwatch sensor data acquisition remain limited. To bridge this gap, SensorW was developed, an open-source Wear operating system (OS)-based framework for direct lifelog data collection from smartwatches without requiring a paired smartphone or manufacturer cloud service. SensorW includes a front-end Wear OS application for sensor data collection and a back-end server for application initiation, secure data submission, and storage. The front-end application uses battery-management strategies, such as hybrid Global Positioning System sampling and wake-lock management, to continuously collect multimodal sensor outputs, including location data, physical activity–related metrics, barometric pressure, and device wear status, and submit data to the back-end server. A preliminary technical evaluation of SensorW was conducted with seven participants who wore SensorW-enabled devices continuously for at least seven consecutive days. The technical evaluation demonstrated multimodal data collection during 17–42 days of monitoring, with operational data availability ranging from 0.73 to 1.00 across participants. These findings support the technical feasibility of SensorW as a practical and extensible platform for continuous digital phenotyping using consumer-grade smartwatches.

## 1. Introduction

### 1.1. Data Collection for Digital Phenotyping

Digital phenotyping has rapidly become a prominent research paradigm in medicine and health sciences, aiming to derive objective and fine-grained representations of individuals’ health and behavioral states through continuous sensing with personal digital devices [1,2]. By leveraging multimodal data streams such as physical activity, physiological signals, environmental context, and behavioral indicators collected from wearable and mobile devices, including smartphones and smartwatches, digital phenotyping enables real-world longitudinal monitoring beyond conventional clinical and epidemiological settings. This approach reduces reliance on self-reported data and mitigates the potential biases associated with conventional survey-based data collection methods [3]. In practice, reliable digital phenotyping requires continuous and energy-efficient sensing frameworks capable of maintaining data acquisition during routine daily use.

### 1.2. Existing Data Collection Frameworks Based on Smartphones

With the widespread adoption of smartphones, several open-source frameworks have been developed to support large-scale mobile-sensor data collection. Beiwe, a well-known smartphone-based sensing platform developed by the Onnela Lab at the Harvard T. H. Chan School of Public Health, was designed to facilitate digital phenotyping research in psychiatry [4]. Similarly, the AWARE framework created by Ferreira et al. at the University of Oulu’s Department of Computer Science and Engineering provides a flexible platform for smartphone-based sensor data acquisition [5]. These frameworks enable researchers to collect data from embedded sensors such as accelerometers and the Global Positioning System (GPS), as well as system-level log data, including phone calls, text messages, network connectivity, Bluetooth activity, audio recordings, and screen status [4,5,6]. By leveraging the sensing capabilities of modern smartphones, these platforms have significantly advanced mobile health and behavioral research.

Numerous epidemiological and clinical studies have used the Beiwe and AWARE frameworks for large-scale mobile-sensing research. For example, Barnett et al. recruited 17 patients with schizophrenia and deployed the Beiwe application on their personal smartphones, demonstrating that relapse episodes could be predicted based on patterns of smartphone usage [7]. Wright et al. conducted a longitudinal study involving patients with gynecological cancers using the Beiwe application to collect physical activity data and administer ecological momentary surveys to assess symptom severity [8]. Similarly, Berry et al. used Beiwe for continuous phone-based sensor collection and self-reported activity tracking in a long-term observational study targeting patients with amyotrophic lateral sclerosis [9]. In another large-scale study, Brown et al. investigated the relationship between user and avatar bonding and depression risk among 565 participants by using AWARE Lite to monitor their daily smartphone usage behaviors [10]. Collectively, these studies illustrate how smartphone-based sensing frameworks have generated valuable insights into health sciences and digital phenotyping research.

However, existing real-world empirical studies using smartphone-based platforms have primarily focused on basic physical activity metrics (such as step counts), smartphone usage patterns, and self-reported survey outcomes. Smartphones generally lack advanced physiological sensors, such as continuous heart rate monitors, which limits their ability to accurately infer sleep status, metabolic equivalents, and other health-related indicators. These sensing limitations pose challenges for smartphone-based frameworks in capturing fine-grained physiological states, which are becoming increasingly important in epidemiological and clinical research.

### 1.3. Current Difficulties in Wearable Devices Data Collection

The rapid proliferation of wearable technologies, particularly smartwatches, presents an opportunity to address the sensing limitations of smartphone-based frameworks. Numerous digital phenotyping studies have been published using smartwatches [11,12,13,14,15,16]. Compared to smartphones, smartwatches are typically equipped with health-oriented sensors such as heart rate monitors, off-body detection sensors, and skin temperature sensors. These additional sensing capabilities enable the collection of fine-grained and continuous lifelog data, supporting more precise digital phenotyping.

Despite these advantages, platforms for longitudinal sensor data collection from wearable devices remain limited. As illustrated in Figure 1, there are three primary technical approaches to collecting data from smartwatches.

The first and most common approach is cloud-based extraction via device manufacturers’ servers. For example, Google Pixel Watch devices (Google LLC, Mountain View, CA, USA) upload lifelog data to Fitbit cloud servers, and Fitbit provides a web application programming interface (API) that allows developers to retrieve user data. Several commercial platforms rely on the Fitbit Web API to collect lifelog data for research purposes, and researchers may develop custom applications based on this API [17,18]. However, this approach is inherently dependent on the manufacturer-provided APIs. Different smartwatch brands require different extraction tools, which limits interoperability. In addition, as a power-saving strategy, many commercial smartwatches record GPS trajectories only during physical training. Consequently, continuous daily GPS tracking through manufacturer-server-based extraction is often unavailable. The second approach involves indirect extraction of lifelog data using a paired smartphone. Although commercial platforms exist [19], open-source free tools are limited. The NativeScript-WearOS-Sensors project, for example, is one of the few open-source tools supporting this method [20]. This framework collects sensor data on the smartphone by installing a corresponding application on both the smartphone and the Wear OS smartwatch. Although this approach provides a proof-of-concept for smartwatch-based sensing, it requires participants to pair their smartwatches with an Android smartphone. RADAR-base is a comprehensive open-source platform supporting multiple smartphones and wearable devices [21]. Depending on the device, wearable data are acquired through a smartphone gateway, HealthKit, or vendor-provided APIs; thus, smartwatch data acquisition is generally not implemented as an autonomous watch-to-server pathway. Similarly, mCerebrum provides a comprehensive Android-based platform for mobile and wearable sensing, with an Android smartphone serving as the central gateway for data acquisition and communication with wearable sensors [22]. Cozie is an open-source research platform that combines smartwatch-based ecological momentary assessment with physiological and contextual data collection [23]. Its Apple Watch (Apple Inc., Cupertino, CA, USA) implementation enables participants to complete micro-surveys directly on the watch and incorporates physiological data retrieved through Apple HealthKit. The platform is designed around a paired iPhone (Apple Inc., Cupertino, CA, USA)–Apple Watch configuration, with data transmission occurring after synchronization with the iPhone. Although several of these platforms provide established infrastructures for longitudinal sensing, their reliance on smartphones or device-specific data interfaces may limit their use in populations in which ownership or management of a compatible smartphone cannot be assumed. The third approach involves direct data extraction from smartwatches. WEARDA is an open-source application that follows this approach and enables independent acquisition of raw sensor data on a consumer smartwatch [24]. However, WEARDA was developed for the Samsung Gear Fit2 Pro (Samsung Electronics Co., Ltd., Suwon, Republic of Korea) running Tizen OS, an operating system that is no longer widely used in current consumer smartwatches. Moreover, the collected data are stored locally on the smartwatch and subsequently transferred to a researcher’s computer rather than being automatically transmitted to a remote research server. These existing systems illustrate the range of architectures available for mobile and wearable sensing. However, among the open-source systems reviewed here, we did not identify a platform that simultaneously provides native Wear OS–based multimodal sensing, operation independent of a paired smartphone and manufacturer cloud services, and direct data transmission from the smartwatch to a researcher-controlled server. To address this architectural gap, SensorW was developed in this study, a Wear OS–based open-source application designed to enable direct and continuous lifelog data collection from smartwatches without requiring a paired smartphone or manufacturer cloud service. Table 1 summarizes the key architectural characteristics of existing open-source mobile and wearable sensing platforms relevant to smartwatch-based data collection.

### 1.4. Motivation and Objectives

This work was motivated by the Iwaki Health Promotion Project (IHPP), an ongoing large-scale community-based annual health-screening program conducted in Aomori Prefecture, Japan. Since its initiation in 2005, the IHPP has enrolled approximately 1000 adult residents annually and contributed to local health promotion and quality-of-life improvement initiatives. Comprehensive health examinations include assessments of physical function, ophthalmological evaluations, blood tests, mental health surveys, microbiome analysis, and other multidimensional measurements, resulting in high-dimensional, large-scale longitudinal datasets [25]. To further expand its data modalities, the IHPP plans to integrate smartwatch-based lifelog data into future health checkups. Long-term continuously recorded behavioral and physiological data, including social participation (inferred from GPS trajectories), daily physical activity (such as step count and caloric expenditure), sleep-related measures, and heart rate (beats per minute (BPM)), will be linked with existing IHPP clinical and survey data to support advanced digital phenotyping research.

Achieving this integration requires a reliable smartwatch-based sensing framework designed to support prolonged data collection during daily use. The primary objective of this study was to develop SensorW, an open-source framework for smartwatch-based sensor data collection tailored to the IHPP lifelog initiative. By providing transparent data acquisition algorithms and an openly accessible system architecture, SensorW promotes methodological reproducibility in empirical research, unlike commercial cloud-based extraction services where data-processing pipelines are often opaque to researchers. Although designed to meet the specific requirements of the IHPP, this framework is intended to serve as a flexible and extensible platform for broader wearable-based health research.

## 2. Materials and Methods

The SensorW framework (version 1.0.0) consists of two main components: a front-end client and back-end server. The front end is implemented as a Wear OS application that runs as a foreground service, enabling continuous sensor data collection and transmission to the server. The back-end server is deployed on a system running Rocky Linux 10 and provides Hypertext Transfer Protocol Secure (HTTPS) services for application initialization and for receiving sensor data uploaded by client devices. Researchers can verify data uploads through server-side logs. The overall workflow for data acquisition and transmission is shown in Figure 2. The SensorW framework was developed using the Java programming language in the Android Studio integrated development environment (version 2024.3.2 Patch 1; Google LLC, Mountain View, CA, USA).

### 2.1. Front-End for Sensor Data Collection and Submission

After installation, the SensorW application requires the user to enter the uniform resource locator (URL) and authentication token of the study server during initialization. The application attempts to establish a connection with the server; upon successful communication, it starts and maintains a foreground service with a notification channel for continuous data tracing.

As the application is designed for long-term lifelog data collection, a system broadcast receiver is registered to capture the device boot-completion event. When a device is rebooted, the application automatically restarts the foreground service to resume data collection. In addition, if the foreground service is unexpectedly terminated, the application sends a notification requesting the user to restart the tracing process manually. These mechanisms are intended to maintain the persistence of foreground services and reduce interruptions during prolonged data collection.

For location data acquisition, the application relies on Google Play services Location APIs (play-services-location version 21.3.0; Google LLC, Mountain View, CA, USA) to obtain information such as latitude, longitude, altitude, horizontal location accuracy, vertical location accuracy, speed, speed accuracy, and whether the location observation is a mock location. Fine location permission is required, not coarse location permission. By default, the sampling interval for location data is set to once every 90 s. When the smartwatch is paired with the user’s Android smartphone, the application prioritizes retrieving location information from the paired smartphone. Due to the high energy consumption of the GPS sensor, a wake-lock management strategy was implemented to balance data resolution and battery consumption. When the system wake lock is held, the application actively records GPS data at a fixed interval of 90 s, providing higher temporal resolution but potentially increasing energy consumption. When the wake lock is released, the system switches to passive location acquisition mode, in which GPS observations are returned at longer and less regular intervals, as determined by the operating system. This passive mode is intended to reduce energy consumption by decreasing the frequency of active GPS acquisition. The wake lock is released when any of the following conditions are met: (1) the user is detected as sleeping; (2) the device is detected as off-body; (3) the user remains in a passive activity state (i.e., no movement or speed detected) for six consecutive GPS observations; or (4) six consecutive GPS data requests return null results.

In addition to location data, barometric pressure and device wear status are obtained through hardware sensors, specifically a barometer and a low-latency off-body detection sensor. Barometric pressure measurements can be used to estimate altitude because altitude values derived directly from GPS are often inaccurate. The default sampling rate for barometric pressure is every 90 s but may be reduced owing to the wake-lock hold/release strategy. Low-latency off-body detection sensors report on- and off-body events and play an important role in battery-management strategies.

Physical activity–related metrics are obtained through Android Health Services using the Health Services client library (androidx.health:health-services-client version 1.0.0; Google LLC, Mountain View, CA, USA). These include cumulative step count, floors ascended, travel distance, energy expenditure, current heart rate, and activity state. These data are retrieved by registering passive sensor event listeners. As a result, the sampling intervals for these metrics, except for the activity state, are not fixed and depend on system-level updates. In addition, passive Health Services data types are not necessarily delivered at a common update frequency, and their delivery cadence may vary across metrics depending on system-level processing and device state. Activity state changes are typically triggered by transitions between sleep and wake states. According to the definition of Android UserActivityState, four states can be returned: asleep, exercise, passive, and unknown. SensorW does not independently classify sleep or activity states. Therefore, the accuracy of these classifications depends on the underlying Health Services implementation and was not independently assessed in the present study.

The sensor data returned by the system are temporarily stored in memory buffers. To reduce the number of input/output operations, buffered data are written to comma-separated value (CSV) files every 30 min. To prevent data loss, buffered data are flushed into local storage when the onDestroy function of the foreground service is triggered. When the accumulated CSV files exceed a predefined size threshold (150 kB by default, typically corresponding to 1–1.5 days of recording), they are compressed into a ZIP archive (approximately 40 kB) and uploaded to the backend server via HTTPS. SensorW does not require an LTE connection. Data can be uploaded via Wi-Fi or, when the smartwatch is paired with a compatible Android smartphone, through the smartphone’s mobile network connection. Thus, Wi-Fi smartwatch models can be used for participants with compatible Android smartphones without requiring an additional LTE subscription. LTE-capable models and subscriptions are primarily required when independent data transmission outside Wi-Fi environments is necessary. If the device cannot reach the server, the ZIP file is retained in local storage and submitted during the next upload attempt once the file size threshold is reached.

### 2.2. Back-End for Sensor Data Receiving and Storage

The backend follows a layered architecture consisting of the network access, reverse proxy, application gateway, and application logic layers. The backend component of the SensorW framework is responsible for application initialization, study protocol delivery, and receiving lifelog data uploaded by client devices. The server is deployed in a Rocky Linux 10 environment and provides secure communication with client applications through HTTPS.

A Cloudflare Tunnel (version 2026.6.0; Cloudflare, Inc., San Francisco, CA, USA)-based network address translation traversal mechanism was used to enable secure access from external networks without directly exposing the internal server. Incoming requests from client devices are first routed through the Cloudflare edge network and then forwarded to the local server through the tunnel service. Within the local server environment, the Nginx (version 1.30.2) web server functions as a reverse proxy managing incoming HTTP requests and forwarding them to the application layer [26]. The application interface is served by Gunicorn (version 23.0.0), a Python (version 3.12.13) Web Server Gateway Interface (WSGI) server that runs the Flask-based (version 3.1.2) back-end application [27]. The Flask application implements several core functions of the SensorW backend framework [28]. First, it handles application initialization requests from client devices and returns the study protocol required for data collection. Second, it receives compressed lifelog data files uploaded by the client application through HTTPS. The uploaded data are then stored on the server for subsequent processing. The current back-end system supports data reception, storage, and basic verification of data uploads through server-side logs. It does not currently include real-time data visualization, participant-level dashboards, or automated alerts. The backend architecture is designed to support secure communication, request handling, and data ingestion for longitudinal lifelog data collection.

All code for constructing the front-end application, along with a step-by-step guide for back-end server deployment, is available on the SensorW project GitHub repository (version 1.0.0; https://github.com/Tadokorokj/SensorW, accessed on 20 June 2026).

### 2.3. Application Setup and Initiation

The application setup and initiation procedure of SensorW is illustrated in Figure 3.

Before data collection begins, users are instructed to grant the required permissions, including fitness and wellness access, location access, notifications, and access to physical activity. To support the automatic resumption of tracing after a device reboot, fitness and wellness access, as well as location access, should be granted at all times, rather than only while the application is in use. In addition, the system option that pauses app activity when the application is inactive should be disabled to reduce the risk of interruptions during prolonged tracing.

After permission configuration, the user proceeds to initialization by entering the server URL, assigned participant ID, and authentication token provided by the research team. The application then attempts to connect to the backend server and retrieve the study information. If a connection is successfully established, the study title, a brief introduction to the study, and the principal investigator’s contact information are displayed on the consent screen. If the application is unable to connect to the backend server, an error message is displayed. After the user reviews the study information and begins tracing, the application enters the continuous data collection phase.

During the tracing period, opening the application interface displays the study information along with the contact details of the research team, allowing participants to identify the responsible investigators and contact them if necessary. In the event of an unexpected application crash, SensorW pushes a notification to alert participants. When the participant reopens the application, a restart screen is displayed instructing the user to manually restart tracing. This design is intended to facilitate recovery after unexpected application termination and reduce the risk of prolonged data interruptions.

### 2.4. Empirical Assessment

To assess the technical feasibility and operational performance of SensorW during daily use, a technical evaluation was conducted with seven research team members. Beginning in February 2026, each participant was instructed to continuously wear a smartwatch running the SensorW application for at least seven consecutive days, with individual start dates varying among participants. The actual observed monitoring durations were 17 to 42 days. Participants were instructed to wear the device during daily activities and sleep, removing it only when there was a risk of device damage (e.g., during showering or bathing) or when charging was required. Of the seven devices used, six were Google Pixel Watch 3 LTE models, and one was a Google Pixel Watch 4 LTE model (both manufactured by Google LLC, Mountain View, CA, USA). The devices ran Wear OS 6. The resulting lifelog dataset was used to characterize data continuity, operational data availability, prolonged data gaps, and empirical sampling yields during daily use. Particular attention was given to the continuity of data collection and the observed data yields of sensor variables acquired under passive sampling or combined passive–active sampling strategies. In addition, participant-level monitoring duration, the number and duration of prolonged data gaps, and operational data availability were calculated. Because the current version of SensorW does not record system-level information on device shutdowns, operating-system process termination, charging, or reboot events, the specific causes of individual data interruptions could not be determined. Therefore, a prolonged data gap was operationally defined as an interval exceeding 2000 s during which no data were recorded from any sensing modality. This threshold was selected to distinguish prolonged interruptions from ordinary variation in data-delivery intervals and was substantially longer than the nominal acquisition intervals of the regularly acquired modalities. Charging-related and other causes of prolonged gaps could not be distinguished retrospectively. A sensitivity analysis was additionally conducted using thresholds of 1000, 3600, and 7200 s. Operational data availability for each participant was calculated using the following equation:

$$Operational\;data\;availability=1-\frac{\sum \left(duration\;of\;prolonged\;data\;gaps\right)}{observed\;monitoring\;span}$$

Because the timing of Health Services–derived passive measurements is controlled by Wear OS and no fixed expected number of observations is defined, conventional sample-level completeness could not be calculated for these modalities. For each sensing modality, the pooled observed data yield was calculated as the total number of eligible observations across all participants divided by the total eligible observation time in person-hours. Participant-specific yields were calculated analogously, and their interquartile ranges (IQRs) were reported. For non-GPS modalities, eligible observation time excluded prolonged data gaps. For GPS, eligible observation time additionally excluded periods when the device was off-body or the participant was classified as asleep; overlapping excluded intervals were merged before their durations were summed.

Data cleaning, organization, and summary analyses were performed using R (version 4.4.2; R Foundation for Statistical Computing, Vienna, Austria) [29]. Data manipulation was conducted with the dplyr package (version 1.2.0) [30]. Non-GPS variables were visualized using ggplot2 (version 4.0.2) [31], whereas GPS trajectory data were visualized using Python (version 3.9.13). Note that the Python script generates the underlying GPS trajectory visualization rather than the final annotated Figure 4c. De-identified non-GPS sensor data collected during the technical evaluation are available in a publicly accessible GitHub repository. The analysis and visualization code is available in the Supplementary Materials.

## 3. Results

The SensorW application developed in this study is available in the SensorW project GitHub repository (https://github.com/Tadokorokj/SensorW, accessed on 20 June 2026), where the source code can be freely accessed.

Table 2 summarizes the observed data yields during empirical assessment. Overall, the results indicated that SensorW successfully captured multiple data modalities, including location, physical activity–related metrics, barometric pressure, and device wear status during the technical evaluation. Pooled observed data yields were calculated using the total number of eligible observations divided by the total eligible person-hours across participants.

For location data, the observed data yield was 24.23 observations per hour when the device was on the body and the participant was awake. Although the nominal location request interval was set to once every 90 s (theoretically 40 observations per h), the observed yield was lower than this nominal maximum. This difference is consistent with the hybrid GPS acquisition strategy, under which active GPS acquisition is reduced when the wake lock is released.

For physical activity–related variables, step count, distance, energy expenditure, floor ascension, and heart rate were recorded at observed data yields of 8.47, 21.89, 23.49, 11.88, and 67.93 observations per h, respectively. The corresponding participant-level IQRs are presented in Table 2. Because these variables were obtained through passive Android Health Services listeners, observations were delivered at system-determined and potentially irregular intervals rather than at a fixed sampling frequency. In particular, the heart-rate data yield should not be interpreted as uniformly spaced measurements at approximately 60 s intervals, as observations may be temporally clustered when delivered by the operating system. These findings indicate that SensorW can continuously obtain passive updates for key behavioral and physiological indicators during daily use. In addition, barometric pressure was recorded at an observed data yield of 26.43 observations per h. In contrast, activity and device wear statuses were not summarized. These two indicators are reported as event-triggered categorical state changes rather than as regularly sampled continuous measurements. Specifically, activity status changes are typically triggered by transitions in the participant’s wake state, whereas device-wear status changes are triggered when the smartwatch is worn or removed.

Table 3 summarizes participant-level observed monitoring duration, prolonged data gaps, and operational data availability during the technical evaluation. Observed monitoring duration ranged from 17.38 to 41.87 days across the seven participants. The number of prolonged data gaps ranged from 0 to 11 per participant, and the total duration of these gaps ranged from 0 to 8.23 days. Operational data availability ranged from 0.73 to 1.00. One participant had no prolonged data gaps and achieved 1.00 operational data availability. These results provide a participant-level description of data continuity during the technical evaluation rather than evidence of long-term system reliability. Pooled operational data availability was 0.896, calculated as one minus the total duration of prolonged data gaps across all participants divided by the total observed monitoring duration across all participants. In a sensitivity analysis, pooled operational data availability was 0.895, 0.896, and 0.899 when the threshold for defining a prolonged data gap was set to 1000, 3600, and 7200 s, respectively.

Figure 4 presents representative lifelog data collected by SensorW during the technical evaluation. Figure 4a,b show time series of activity-related metrics over a 24-h period, including energy expenditure, heart rate (BPM), step count, and cumulative travel distance. The gray and pink shaded areas indicate periods when the device was detected as off-body and when the participant was classified as being in an asleep state by Android Health Services, respectively. Figure 4c provides an example of the GPS trajectory recorded by SensorW during the participant’s sightseeing visit in Kumamoto City, Japan. The displayed segment was deliberately selected because it showed a readily interpretable sightseeing trajectory in a public area and did not disclose the participant’s residence, workplace, university, usual commuting route, or routine daily mobility pattern. The participant departed from the starting location indicated by the red point at the bottom of the map, traveled toward the area highlighted by the red ellipse along the direction indicated by the red arrows, and subsequently returned along the direction indicated by the blue arrows. The participant reviewed the exact map and provided written consent for its publication. These examples illustrate the technical feasibility of collecting multimodal lifelog data with SensorW during everyday use.

Taken together, these results support the technical feasibility of SensorW for continuous multimodal lifelog data collection during daily use.

## 4. Discussion

The present technical evaluation provides an initial characterization of the operational performance of SensorW during daily use. Across the seven participants, SensorW collected multiple behavioral, physiological, and contextual data modalities, while participant-level operational data availability ranged from 0.73 to 1.00. For GPS, the pooled yield was approximately 24.23 observations per eligible person-hour, compared with a nominal maximum of 40 observations per hour when active 90 s acquisition was maintained. The difference is consistent with the intended hybrid acquisition strategy, in which SensorW switches to passive location acquisition during sleep, off-body periods, prolonged inactivity, or repeated unsuccessful location requests.

Direct comparison of these sampling yields with those reported for existing platforms such as Beiwe or AWARE should be interpreted cautiously. Sampling performance in mobile and wearable sensing studies depends on the configured sampling protocol, hardware and operating-system environment, power-management strategy, participant adherence, and the definition of eligible monitoring time. Moreover, Beiwe and AWARE are primarily smartphone-based sensing frameworks, whereas SensorW was designed for direct Wear OS–based sensing. Therefore, the present results are better interpreted as a characterization of SensorW under the evaluated configuration than as evidence of superior sampling performance relative to existing platforms. Evaluation in a larger and more heterogeneous population will be necessary to characterize its field performance under population-level deployment conditions.

Nevertheless, several limitations of this study require further investigation. First, battery performance was not objectively quantified in the present technical evaluation because the current version of SensorW did not log battery-level information. Consequently, the energy-saving effect of the hybrid GPS sampling and wake-lock management strategies could not be directly evaluated. Future studies will incorporate continuous battery-level logging and comparative testing under different GPS acquisition and wake-lock conditions. Further optimization of wake-lock management and GPS sampling algorithms is needed to extend battery life under more demanding conditions. Improving the balance between sensing precision and energy consumption remains an important area for future development. Second, to limit energy consumption, the current version of SensorW primarily supports passive data collection for physical activity–related metrics and heart rate. Some research scenarios may require higher sampling rates or more uniform sampling intervals, particularly for heart rate data, which are increasingly used for cardiovascular risk prediction [32]. Similarly, although accelerometer data are technically accessible, continuous high-frequency accelerometer recordings are highly energy-consuming when written to external storage. Consequently, the current implementation does not support raw accelerometer data logging. Wearable accelerometer data is a valuable resource for real-world gait analysis and mobility assessment [33]. Providing configurable options for sampling frequency, accelerometer logging, and advanced physiological monitoring will be key objectives for future versions to support diverse study designs. Third, owing to budget constraints, the present technical evaluation was conducted only on Google Pixel Watch 3 and 4. Different smartwatch manufacturers may implement proprietary foreground service management and background execution policies, which could affect the application stability and automatic restart mechanisms. Furthermore, sensor availability varies across smartwatch models. For example, some low-cost devices do not include dedicated low-latency off-body detection sensors, requiring the development of more robust and generalized off-body detection mechanisms. Future work will, therefore, extend compatibility testing across additional smartwatch brands and models to improve robustness and generalizability. Fourth, although SensorW implements automatic restart mechanisms and fault-tolerant data transmission strategies, real-world deployments may still encounter unexpected operating system restrictions, storage interruptions, or network instabilities that could result in temporary data loss. Further strengthening of system resilience and long-term stability under heterogeneous field conditions will be an important direction for improvement. Fifth, system-level event logs were unavailable; therefore, specific application crashes and subsequent recovery outcomes could not be identified retrospectively. Consequently, application stability and crash-recovery performance could not be directly evaluated in the present study. Incorporating system-level event logging to capture application termination, device reboot, and related recovery events will be an important direction for future development. Sixth, sleep and activity-state classifications were obtained directly from Android Health Services and were not validated against an independent reference measure. Consequently, the recorded “asleep” state should not be interpreted as a validated measurement of sleep duration or sleep quality. Future studies should assess the agreement of these classifications with validated sleep measures before their use as primary phenotyping variables. Seventh, the present technical evaluation involved only seven research team members and therefore represents a small convenience sample. The findings cannot establish long-term reliability or generalizability to heterogeneous user populations.

In addition, SensorW will be deployed in the upcoming Iwaki Health Promotion Project to evaluate its performance in a large-scale real-world digital phenotyping study. Feedback from participants and field researchers will inform the iterative refinement of system functionality and user interface design. Finally, the development of a data visualization and monitoring platform, potentially implemented as an R Shiny–based web application, represents another important direction for enhancing research usability and real-time oversight [34].

## 5. Conclusions

In this study, SensorW was developed as an open-source Wear OS–based wearable sensing framework designed to support longitudinal digital phenotyping research. SensorW enables researchers to initiate continuous multimodal data collection through a streamlined deployment procedure, facilitating real-world lifelog acquisition using consumer-grade wearable devices. This preliminary real-world technical evaluation, lasting 17–42 days per participant, demonstrated the feasibility of collecting multimodal lifelog data, including GPS location, heart rate, sleep status, step count, floor count, distance, caloric expenditure, and barometric pressure. Further evaluations involving larger and more diverse populations and longer observation periods are required to establish long-term reliability and robustness. Although certain technical and deployment limitations remain, we plan to extend the SensorW framework by incorporating data visualization and real-time monitoring modules and enhancing its scalability and usability. We envision SensorW as a flexible and accessible platform to support research teams worldwide in conducting large-scale digital phenotyping studies.

## Figures and Tables

**Figure 1:**
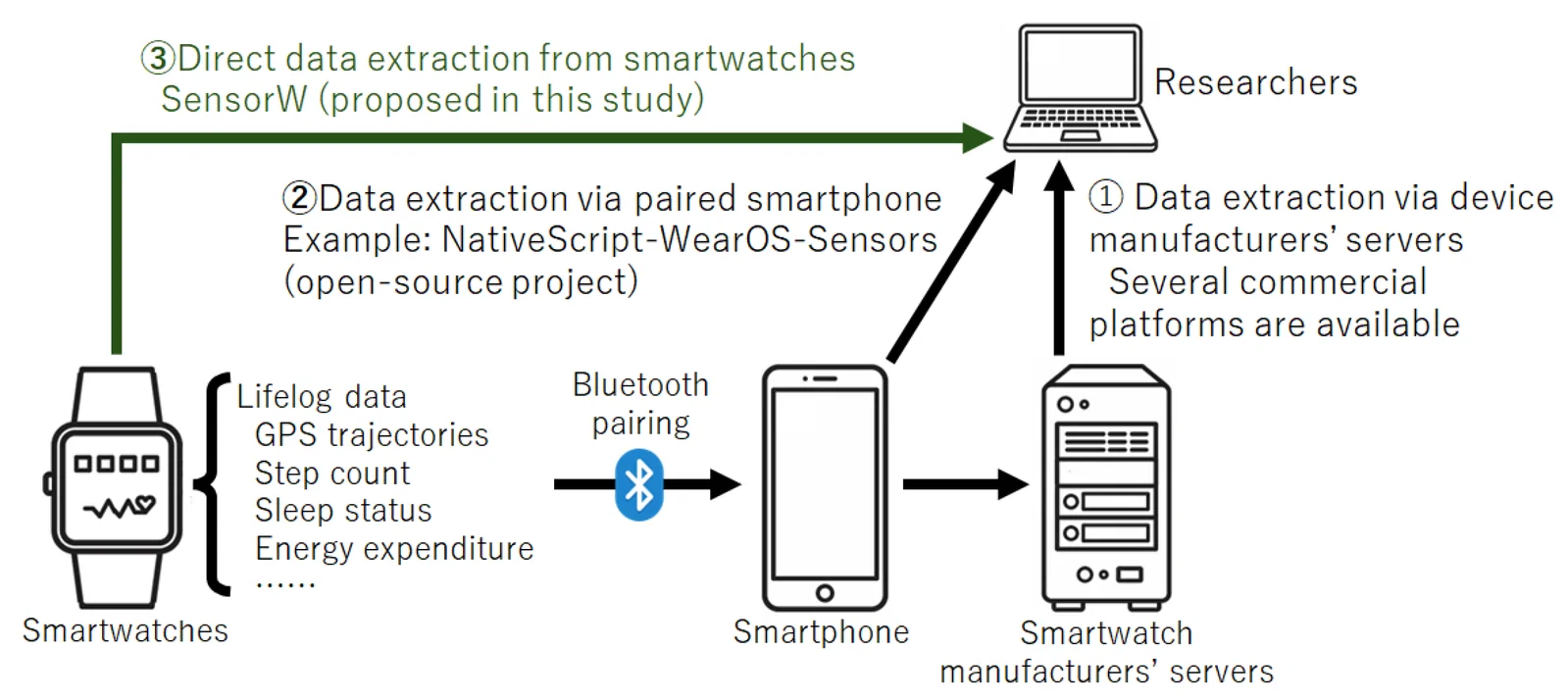
Existing and proposed approaches for smartwatch lifelog data collection. Three possible approaches for collecting lifelog data from smartwatches are illustrated. 1. Cloud-based extraction via device manufacturers’ servers (commonly used in commercial platforms). 2. Indirect extraction via paired smartphones (e.g., NativeScript-WearOS-Sensors). 3. Direct data extraction from smartwatches to researcher-controlled servers, as proposed in this study (SensorW).

**Figure 2:**
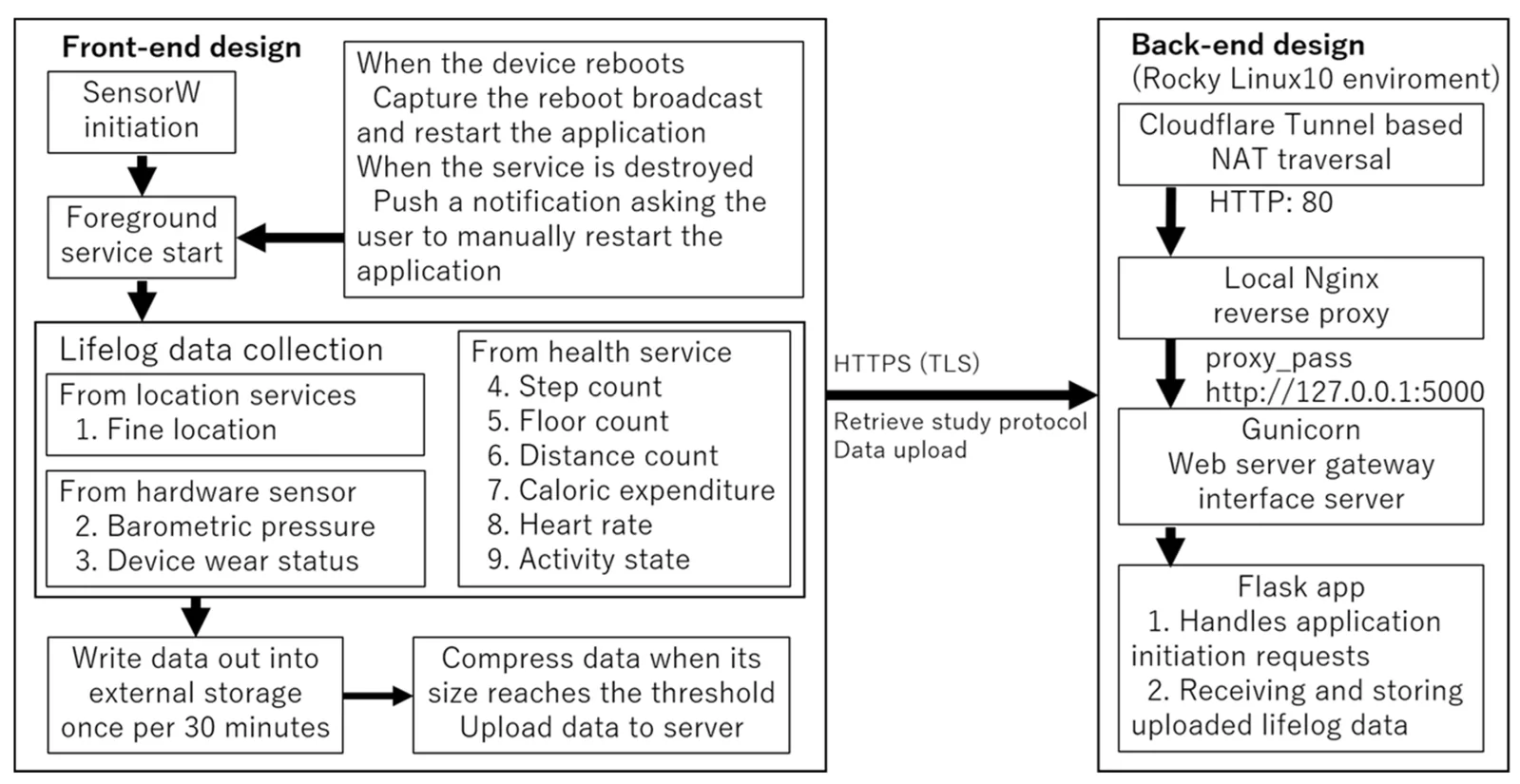
Architecture of the SensorW framework. The SensorW framework consists of a front-end Wear OS application for sensor data acquisition and transmission, and a back-end server that delivers study protocols and receives uploaded lifelog data from client devices. Black arrows indicate the sequence and direction of processing steps within the front-end and back-end workflows. SensorW records location data, physical activity–related metrics, barometric pressure, and device wear status, which are then transmitted to the research server for persistent storage and further analysis.

**Figure 3:**
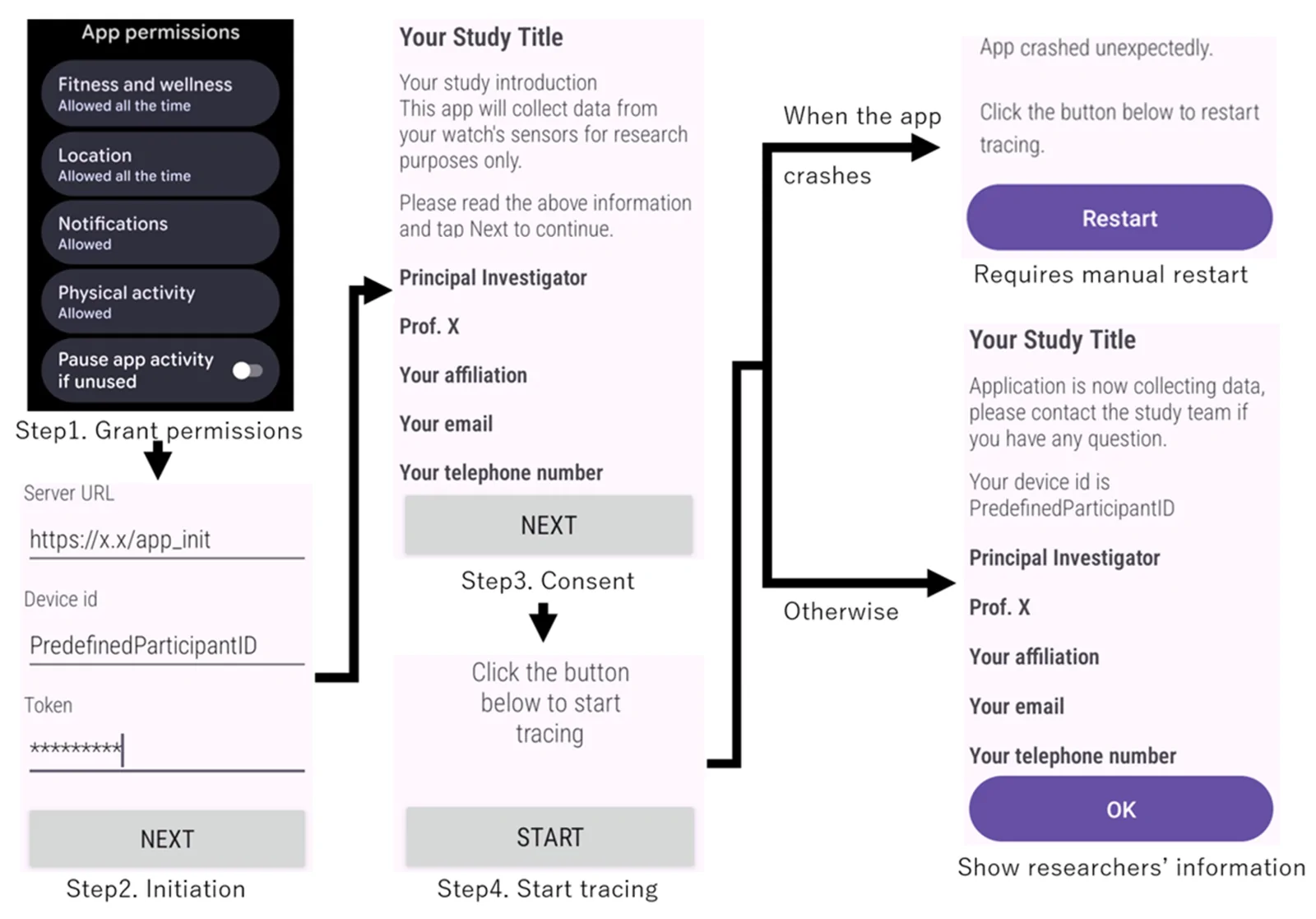
Workflow for initiating the SensorW application. After installation and permission authorization, the users must supply the server URL, assigned participant ID, and an authentication token to initialize the application. Asterisks indicate masked characters of the authentication token. The application then connects to the backend server to retrieve the study protocol. After consent is obtained, SensorW begins continuous lifelog data collection.

**Figure 4:**
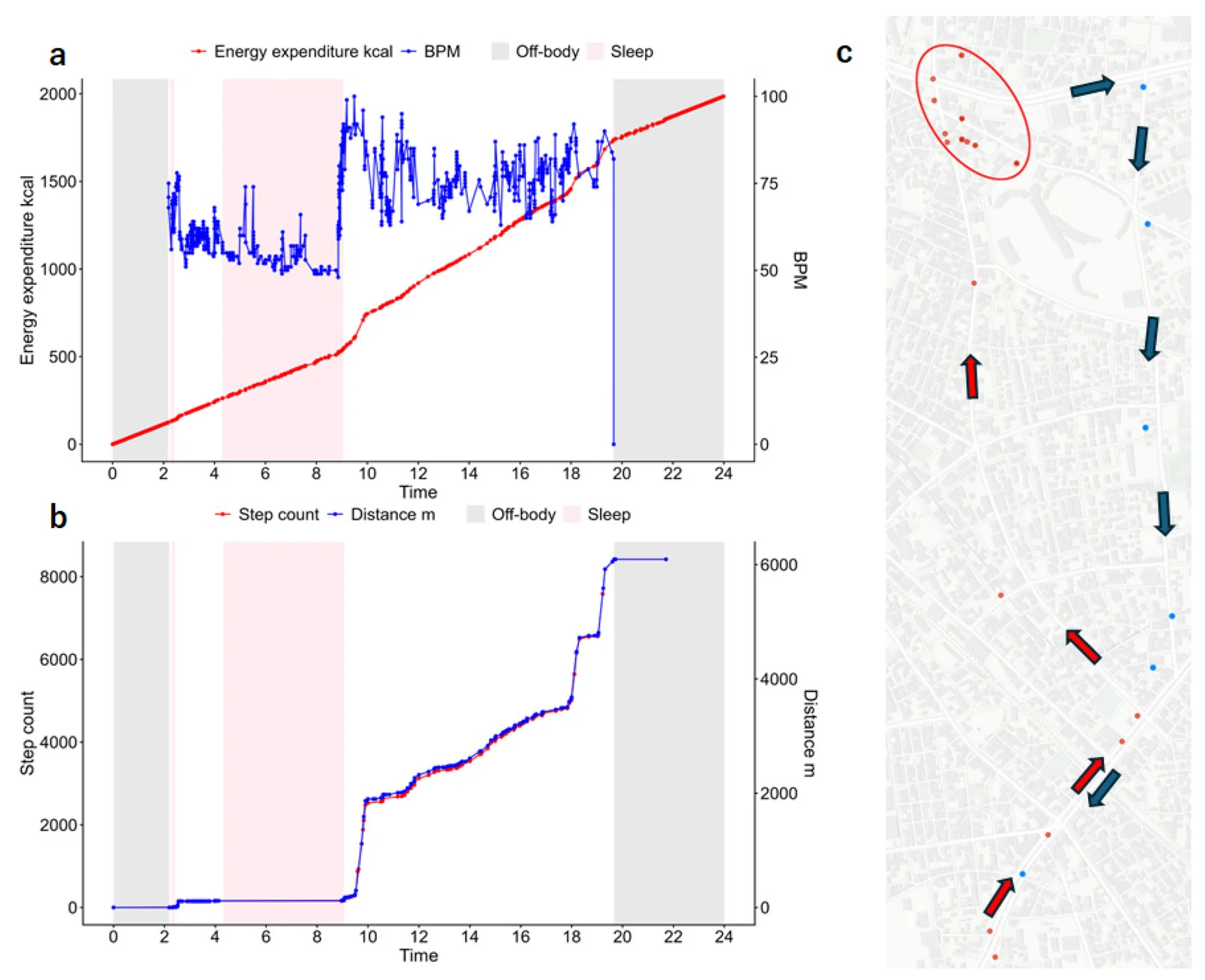
Example lifelog data collected by SensorW during the technical evaluation. (**a**) Time series of energy expenditure and heart rate over a 24-h period. (**b**) Time series of step count and cumulative travel distance over the same period. Gray and pink shaded areas indicate periods when the device was off-body and periods when the participant was classified as asleep by Android Health Services, respectively. (**c**) Deliberately selected GPS observations recorded during a sightseeing visit in Kumamoto City, Japan. It does not include the participant’s residence, workplace, university, usual commuting route, or routine daily mobility pattern. Red and blue dots indicate GPS observations recorded during the outbound and return portions of the trajectory, respectively. The arrows indicate the direction of movement, and the red ellipse highlights an area in which multiple GPS observations were recorded. The directional arrows and red ellipse were added manually for illustration.

**Table 1:** Comparison of open-source mobile and wearable sensing platforms relevant to smartwatch data collection.

Project (License)	Smartwatch Support	Phone Dependency	Manufacturer Cloud Dependency	Representative Data Modalities
SensorW (Apache-2.0)	Wear OS	No mandatory paired phone	No	GPS, heart rate, steps, distance, energy expenditure, sleep/off-body, etc.
AWARE/ AWARE-Light (Apache-2.0) [5,6]	Primarily smartphone; external-device integration available	Yes	Depends on integrated external source	GPS, accelerometer, gyroscope, magnetometer, barometer, device/context data, surveys, etc.
Beiwe (BSD-3-Clause) [4]	No native smartwatch sensing	Yes	No	GPS, accelerometer, gyroscope, call/text metadata, microphone, surveys, etc.
NativeScript- WearOS-Sensors (Apache-2.0) [20]	Wear OS	Yes	No	Accelerometer, gyroscope, magnetometer, heart rate, GPS
RADAR-base (Apache-2.0) [21]	Broad wearable/mobile support	Generally phone mediated for wearable sensing	Device dependent	Phone sensors; activity, heart rate, sleep, peripheral oxygen saturation, temperature and device-specific wearable streams
mCerebrum (BSD-2-Clause) [22]	Android smartphone- centered platform integrating wearable sensors	Yes	No	Smartphone and high-frequency wearable sensor streams; derived biomarkers
Cozie (LGPL-3.0) [23]	Apple Watch	Yes	No	Micro-surveys, heart rate, activity, sound and HealthKit-derived data
WEARDA (MIT) [24]	Tizen OS	No	No	Raw smartwatch sensor data

Smartwatch support refers to native smartwatch sensing or wearable-device integration. Phone dependency indicates whether a paired or gateway smartphone is required for wearable data acquisition or transmission. Manufacturer cloud dependency refers specifically to reliance on manufacturer-operated cloud services or vendor cloud APIs and does not include researcher-controlled or self-hosted servers.

**Table 2:** Observed data yields during the technical evaluation.

Lifelog Data		Sensor	Observed Data Yield (Observations/Person-Hour), Pooled Rate (Participant-Level IQR)
Location	Latitude	GPS	24.23 (5.29), when the device is on-body and participant is awake
	Longitude		
	Altitude		
	Horizontal location accuracy		
	Vertical location accuracy		
	Speed		
	Speed accuracy		
	If the data is mock		
Physical activity	Step count	Accelerometer, heart rate sensor, GPS, barometer	8.47 (5.17)
	Distance (m)		21.89 (15.88)
	Energy expenditure (kcal)		23.49 (3.45)
	Floors ascended		11.88 (10.67)
	Beats per minute		67.93 (38.32)
	Activity status (asleep or passive)		-
Barometric pressure (hPa)		Barometer	26.43 (2.76)
Device wear status		Low-latency off-body detection sensor	-

The device reported activity status as a categorical state. The application records the activity state reported by Android Health Services. Similarly, the device switches between on- and off-body states, depending on whether the watch is worn or removed. Event-triggered indicators, such as activity status and device wear status, were not summarized. Values represent pooled data yields calculated as the total number of observations divided by the total eligible person-hours (excluding prolonged gaps) across all participants. Values in parentheses indicate the interquartile range (IQR) of participant-specific yields. For GPS, eligible observation time was restricted to periods when the device was on-body, and the participant was awake.

**Table 3:** Participant-level observed monitoring duration, prolonged data gaps, and operational data availability during the technical evaluation.

ID	Observed Monitoring Duration (Days)	Number of Prolonged Data Gaps	Total Duration of Prolonged Data Gaps (Days)	Median Duration of Individual Prolonged Data Gaps (IQR) (Days)	Operational Data Availability
1	23.77	10	2.16	0.16 (0.15)	0.91
2	34.26	0	0	- (-)	1.00
3	41.87	11	8.23	0.33 (0.72)	0.80
4	18.38	2	1.84	0.92 (0.76)	0.90
5	30.60	4	0.31	0.08 (0.01)	0.99
6	23.20	8	6.34	0.40 (0.79)	0.73
7	17.38	5	0.85	0.10 (0.13)	0.95

A prolonged data gap was operationally defined as an interval exceeding 2000 s during which no data were recorded from any sensing modality. Operational data availability was calculated as 1 − (total duration of prolonged data gaps/observed monitoring span). IQR: interquartile range; -: not applicable because no prolonged data gap occurred.

## Data Availability

The SensorW source code and de-identified multimodal sensor data used in the technical evaluation, excluding participant-level raw GPS coordinates, are available from the publicly accessible GitHub repository (https://github.com/Tadokorokj/SensorW, accessed on 20 June 2026). Raw participant-level GPS coordinates are not publicly released for privacy reasons. The GPS sampling-yield analysis in Table 2 and the trajectory shown in Figure 4c cannot be independently reproduced from the publicly available dataset. The code used for data processing, analysis, and visualization is provided in the Supplementary Materials.

## Supplementary Materials

The following supporting information can be downloaded at: https://www.mdpi.com/article/10.3390/s26196093/s1, Supplementary_codes.zip. The ZIP file includes Python code for visualization of the GPS data and R code for the statistical analyses and generation of figures and tables reported in the manuscript.

## Abbreviations

The following abbreviations are used in this manuscript:

OS	Operating system
GPS	Global Positioning System
API	Application programming interface
IHPP	Iwaki Health Promotion Project
BPM	Beats per minute
HTTPS	Hypertext Transfer Protocol Secure
URL	Uniform resource locator
CSV	Comma-separated value
WSGI	Web Server Gateway Interface
